# Complications Following Nictitating Membrane Resection in Horses: 92 Eyes (2015–2024)

**DOI:** 10.1111/vop.70243

**Published:** 2026-08-06

**Authors:** Tiantian Wu, Darko Stefanovski, Nicole M. Scherrer

**Affiliations:** ^1^ Department of Clinical Studies, New Bolton Center, School of Veterinary Medicine University of Pennsylvania Kennett Square Pennsylvania USA

**Keywords:** equine, neoplasia, ophthalmic, surgery, third eyelid

## Abstract

**Objective:**

The goal of this retrospective study is to evaluate the complications following nictitating membrane resection in horses with the aim of providing valuable information on the risks of this surgical procedure.

**Animals:**

83 horses (92 eyes).

**Procedures:**

Medical records for 83 horses that underwent nictitating membrane resection in 92 eyes between February 2015 and June 2024 were reviewed. Horses were included if they had at least 1 month of follow‐up data available. Owners were contacted via telephone or email questionnaire to obtain follow‐up information. Complications were recorded only when present in the surgical eye and not in the contralateral eye. Owner‐reported findings were included only when they were persistent at the time of follow‐up or noted beyond the immediate postoperative period (> 1 week).

**Results:**

The overall owner reported complication rate was 28.2% (26/92). Complications that occurred within 1 week of surgery were fat prolapse (*n* = 4; 4.3%) and hemorrhage (*n* = 1; 1.1%). Long‐term complications noted by owners included epiphora (*n* = 21; 22.8%) and conjunctivitis (*n* = 9; 9.8%). The incidence of fat prolapse was low, with no cases reported when sutures were placed (*p* = 0.80). There were follow‐up examinations performed for 39 horses and 23.1% had complications. These included epiphora (*n* = 4; 10.3%), conjunctival adhesion (*n* = 1; 2.6%), fat prolapse (*n* = 2; 5.1%), and enophthalmos (*n* = 2; 5.1%).

**Clinical Relevance:**

Overall complications reported by owners and found on examination were minor and easily managed. Chronic mild epiphora is the most common complication that persists after the nictitating membrane resection procedure and should be reviewed with owners.

**Abbreviation**
SCC: squamous cell carcinoma

## 1 Introduction

The nictitating membrane, also known as the third eyelid, serves an important role in ocular health by aiding tear film production and distribution, and globe protection. However, the nictitating membrane is also a common site of ocular neoplasia in the horse [1, 2, 3, 4]. Reported neoplasms that can necessitate nictitating membrane resection include squamous cell carcinoma, lymphosarcoma, hemangiosarcoma, hemangioma, papilloma, basal cell tumor, lymphangiosarcoma, lymphangioma, and adenocarcinoma [3, 5, 6, 7, 8, 9, 10]. Among these, squamous cell carcinoma (SCC) is the most commonly reported neoplasm of the equine nictitating membrane, and horses with lighter pigmentation are at high risk for developing SCC [1, 2, 3, 4]. In these cases, the goal of nictitating membrane resection is to remove the neoplastic tissue in its entirety and prevent recurrence [11, 12].

Compared with corneal or limbal lesions, neoplasms of the eyelid and nictitating membrane demonstrate greater metastatic and local invasion potential due to extensive lymphatic drainage [13, 14]. Complete resection is the most effective treatment for nictitating membrane neoplasia, and delayed removal can lead to extensive local invasion of surrounding tissues and high recurrence rates [3, 15]. In cases of nictitating membrane neoplasia, recurrence has been associated with a high (60%) mortality rate, supporting early and complete surgical resection of the nictitating membrane in affected cases [12].

Although the nictitating membrane contributes significantly to tear film production in dogs, its role in horses appears to be less substantial [11, 16, 17]. According to Labelle 2011, resecting the nictitating membrane was not clinically associated with obvious corneal disease [11]. There are a few studies that describe or report complications following nictitating membrane resection in horses. Long‐term mild discharge was mentioned as a postoperative clinical sign; however, there are no details regarding incidence or severity. Likewise, orbital fat prolapse has been recognized as a potential complication of nictitating membrane resection, and primary conjunctival closure is variably performed. Therefore, the objective of this retrospective study was to characterize postoperative complications following nictitating membrane resection. Findings from this investigation are intended to provide evidence‐based insight into the risks and outcomes of this procedure.

## 2 Materials and Methods

### 2.1 Case Selection

A retrospective review of medical records for horses that underwent nictitating membrane resection for suspected neoplasia between February 2015 and June 2024 was performed. Horses that underwent complete resection of the nictitating membrane, had tissue submitted for histopathologic examination, and had a minimum follow‐up period of 1 month after surgery were included in the study.

### 2.2 Surgical Procedure

**FIGURE 1:**
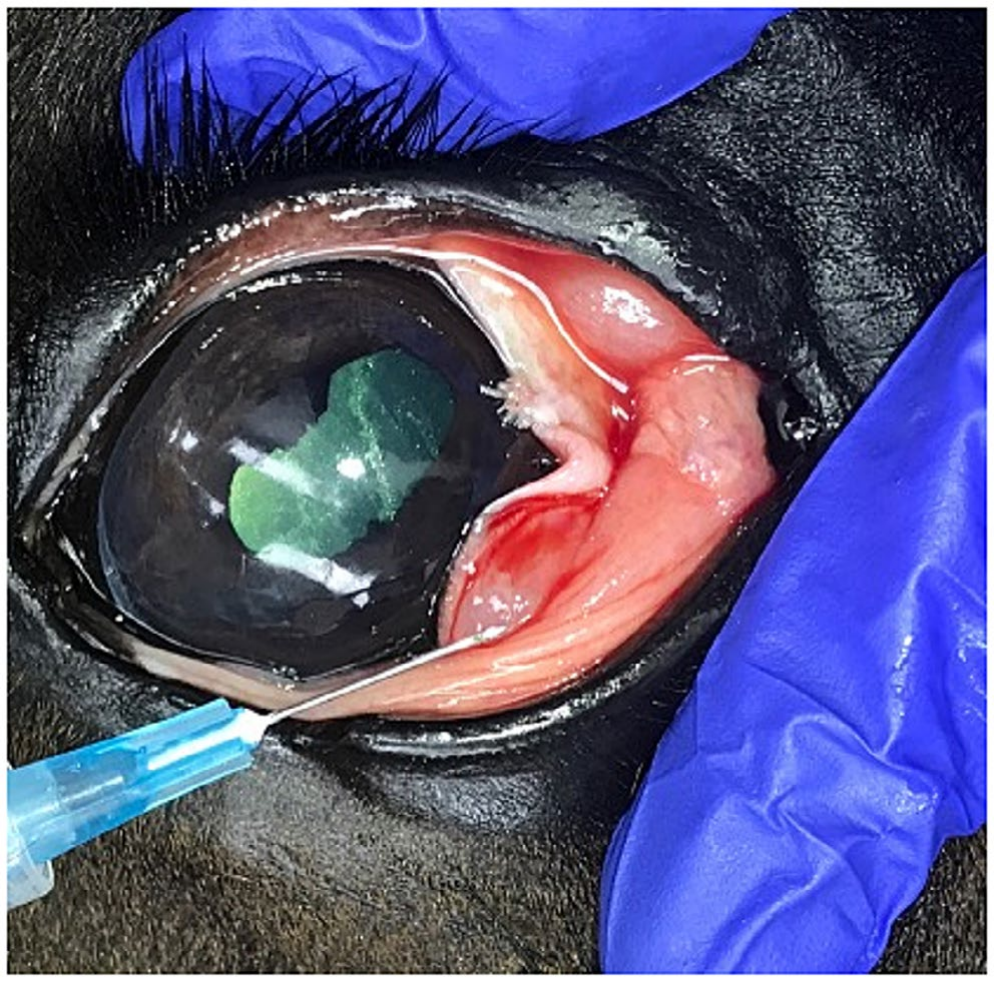
An example of a local anesthetic being instilled at the dorsal edge of the nictitating membrane.

Nictitating membrane resection was performed under either standing sedation or general anesthesia, depending on case and surgeon preference. If nictitating membrane removal was performed as a standing procedure, the body of the nictitating membrane was anesthetized by subconjunctival instillation of local anesthetic (~2 mL mepivacaine, Zoetis, 20 mg/mL) in a V‐shaped area (Figure 1). For procedures performed under either standing sedation or general anesthesia, the nictitating membrane was elevated with forceps in the same V‐shaped area, and the base of the nictitating membrane was clamped with a Kelly hemostat for approximately 1 min, followed by immediate transection with Mayo scissors. This was repeated along the length of the base until the entire nictitating membrane was removed. Suture placement following resection was performed at the discretion of the surgeon. Manual pressure with sterile gauze was applied to the medial canthal area immediately following resection to reduce hemorrhage. All horses received a systemic nonsteroidal anti‐inflammatory (flunixin meglumine, Merck) at 1.1 mg/kg PO q12h for 3 days, followed by 0.5 mg/kg PO q12h for 3 days. In the majority of cases, topical neomycin‐polymyxin B‐bacitracin ophthalmic ointment (Baush and Lomb) was applied postoperatively 2–3 times daily for 1 week. In horses for which topical treatment was not possible, oral trimethoprim sulfamethoxazole (Amneal) for an average of 7–10 days postoperatively was used (30 mg/kg q12h). Resected tissues were submitted for histopathology. Adjunctive procedures, including cryotherapy, radiation therapy, and photodynamic therapy, were not performed in the included cases.

### 2.3 Data Collection

Medical records from the University of Pennsylvania New Bolton Center were reviewed to obtain information on patient signalment (age, sex, breed), eye(s) involved, multiple locations involved (lesions involving the nictitating membrane and additional ocular or periocular structures), standing sedation versus general anesthesia, suture placement, histopathology result, surgical margin, postoperative mask use and type, and follow up examination if available. Histologic diagnosis was categorized as squamous cell carcinoma, conjunctivitis, solar elastosis, lymphofollicular hyperplasia, and other. Mask use was described as no mask worn, < 90% UV light protection worn partially, < 90% UV light protection worn all the time, > 90% UV light protection worn partially, and > 90% UV light protection worn all the time. Phone or email interviews with all owners were conducted to gather data on abnormal clinical signs, current status, and satisfaction rate. To avoid bias, the interviewee completed a complication questionnaire (Additional details are provided in File S1) designed specifically for this study either over the phone or via email. Complications were defined as any abnormal findings observed in the surgical eye following nictitating membrane resection. Complications were recorded only when present in the surgical eye and not in the contralateral eye. While recurrence of neoplasia has been well reported previously in the literature, it was not included as a complication in the current study, as none of the horses underwent confirmatory biopsy of any suspected recurrence. Owner‐reported findings were included only when they were persistent at the time of follow‐up or noted beyond the immediate postoperative period (> 1 week).

### 2.4 Statistical Analysis

All analyses were conducted with Stata 16MP, StataCorp, College Station TX, with two‐sided tests of hypotheses and a *p*‐value < 0.05 as the criterion for statistical significance. Tests of normal distribution (Shapiro–Wilk test) were performed to determine the extent of skewness of all variables. Descriptive analyses included calculation of medians and ranges for continuous variables and tabulation of categorical variables as frequency counts and percentages. Spearman rank pairwise correlation analysis and Firth logistic univariate analysis were used to determine the association between postoperative complications and age, sex, eye affected, one or multiple locations affected, histologic diagnosis, use of sutures after surgical resection, surgical margins, use of topical chemotherapy postoperatively, standing sedation or general anesthesia used, and type of postoperative mask used. The type of complication as a multinomial outcome was analyzed using univariate multinomial logistic regression. All significant univariate associations were reported as a *p* value and the respective odds ratio (OR) unless otherwise specified. Breed distribution in the study cohort was compared with the overall hospital population using a Monte Carlo Chi‐square test after collapsing rare breeds into a single ‘Other’ category. Expected breed frequencies were derived from the hospital database proportions.

## 3 Results

Eighty‐three horses (92 eyes) met the inclusion criteria. Fifty‐four (65%) were male, and 29 (35%) were female. The age of horses ranged from 2 to 28 years (mean ± SD, 14.9 ± 6.3 years; median, 15 years). The right eye was affected in 32 (38.5%) horses, the left eye in 42 (50.6%) horses, and both eyes in 9 (10.8%) horses.

**FIGURE 2:**
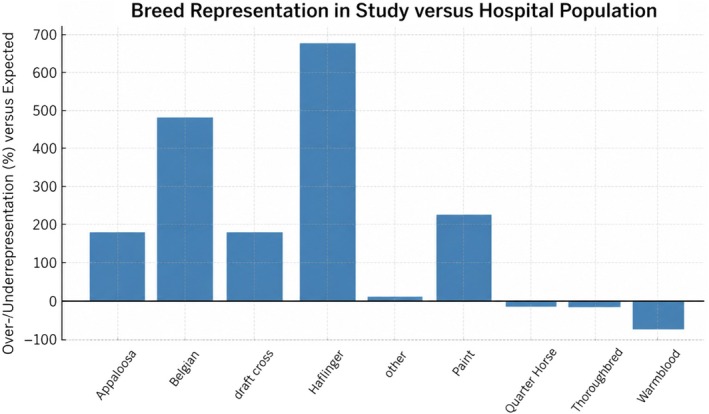
Comparison of breed distribution between study horses (*n* = 93) and the overall hospital population (*n* > 40 000). Bars represent percentage deviation from expected values based on hospital proportions. Positive values indicate overrepresentation; negative values indicate underrepresentation (*p* < 0.001).

Breeds represented included Thoroughbred (*n* = 24), Paint (*n* = 11), Quarter Horse (*n* = 9), Appaloosa (*n* = 7), Haflinger (*n* = 4), Warmblood (*n* = 4), draft cross (*n* = 4), Belgian draft (*n* = 4), Tennessee Walking Horse (*n* = 3), Clydesdale (*n* = 3), American Saddlebred (*n* = 2), appendix Quarter Horse (*n* = 2), mule (*n* = 1), Standardbred (*n* = 1), Oldenburg (*n* = 1), Chincoteague pony (*n* = 1), Percheron (*n* = 1), and Hanoverian (*n* = 1). Breed distribution differed significantly between the study cases and hospital populations (Monte Carlo *c* ^2^ = 82.0, df = 8, *p* < 0.001). Haflingers (+680%), Belgians (+484%), Paints (+226%), Appaloosas (+182%), and draft crosses (+183%) were markedly overrepresented, whereas Warmbloods (−71%) and, to a lesser extent, Thoroughbreds (−17%) and Quarter Horses (−14%) were underrepresented (Figure 2).

**FIGURE 3:**
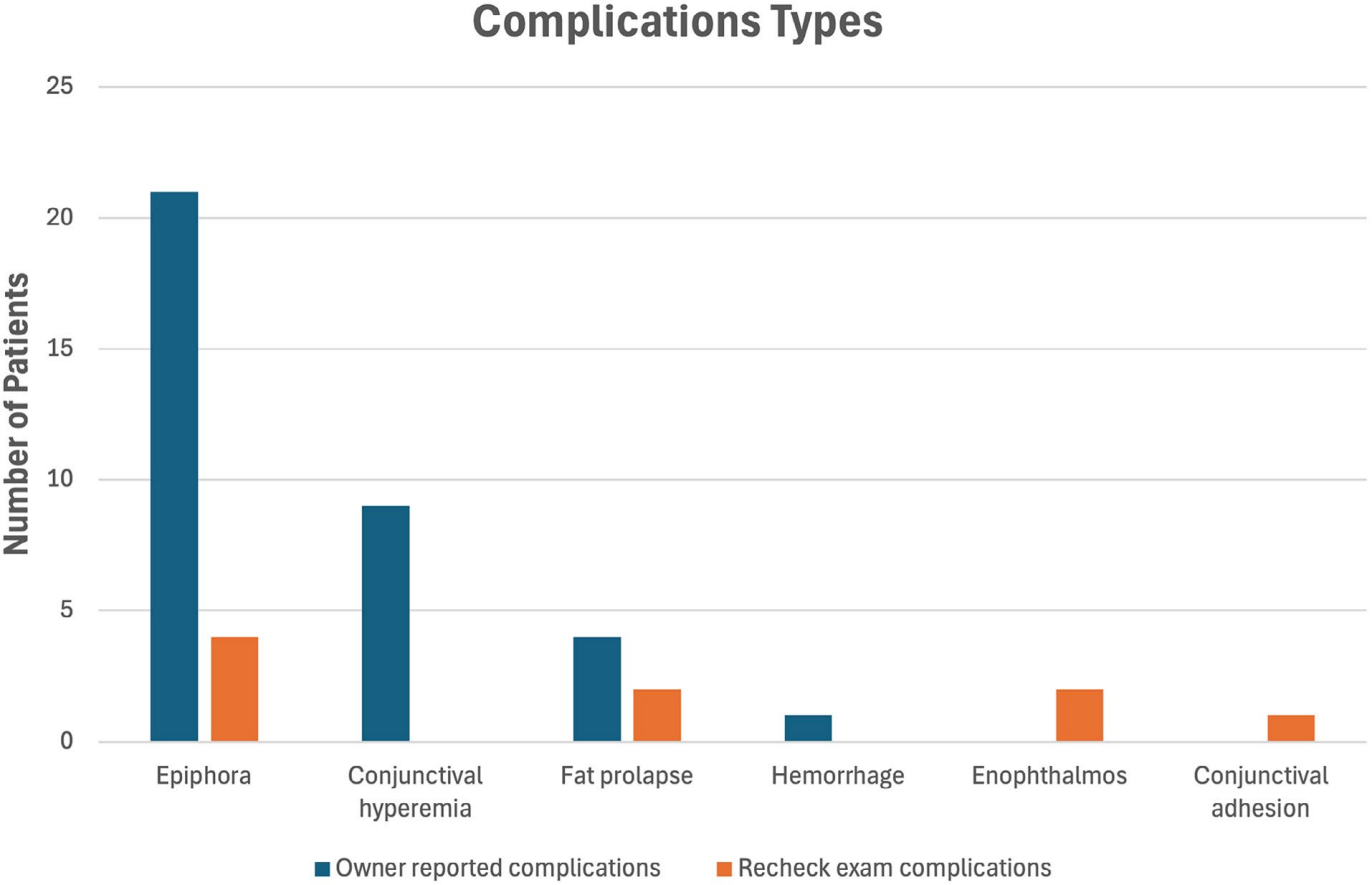
Clustered column bar chart showing the distribution of postoperative complications identified by owner follow‐up interviews and recheck clinical examinations performed by a board‐certified ophthalmologist following nictitating membrane resection.

Owner reported complications occurred in 26 of 92 (28.2%) eyes (Figure 3). Complications that occurred within 1 week of surgery were fat prolapse (*n* = 4; 4.3%), and hemorrhage (*n* = 1; 1.1%). All cases of fat prolapse occurred within 24 h after surgery and resolved with trimming the exposed fat. Long‐term complications noted by owners included epiphora (*n* = 21; 22.8%) and conjunctivitis (*n* = 9; 9.8%). The owner follow‐up period ranged from 1 to 118 months (mean, 37 months; median, 34 months). Despite the presence of persistent complications, owner‐reported satisfaction with the surgical outcome was 100%.

A subset of the horses that underwent nictitating membrane removal returned for recheck examination (*n* = 39). Follow up time ranged from 4 to 1486 days (median 141). Of these horses, complications associated with the surgical eye were reported in 23% (*n* = 9). The only complication that occurred within 1 week of surgery was fat prolapse (*n* = 2; 5.1%). Long‐term complications noted were epiphora (*n* = 4; 10.3%), enophthalmos (*n* = 2; 5.1%), and conjunctival adhesion (*n* = 1; 2.6%).

The only variable associated with owner reported complications was eye affected. Relative to right eye as a reference category, the left eye was associated with a decreased incidence of complications (*p* = 0.043, OR = 0.38). There was no association between owner reported complications and the remaining variables: breed, sex, age, number of affected sites, histologic diagnosis, margins, sutures placed, sedation versus anesthesia, post‐operative topical chemotherapy, post‐operative UV light protection. When performing statistical analysis on the individual owner reported complications (epiphora, hemorrhage, fat prolapse and conjunctivitis) there was no association with any of the investigated variables. Due to the low number of observations for hemorrhage, statistical analysis could not be performed.

Fifty‐nine horses received postoperative topical chemotherapy with 0.04% mitomycin C (SaveWay Compounding Pharmacy) 3–4 times daily for weeks one and three following histopathologic confirmation of SCC. Owners of all horses were advised to use a mask providing > 90% ultraviolet light protection postoperatively. Seventeen owners (17/92; 18.5%) reported partial mask use, primarily during turnout or the summer months; of these, 15 (15/17; 88.2%) used a mask providing > 90% ultraviolet light protection. Forty‐nine owners (49/92; 53.3%) reported continuous mask use, of whom 43 (43/49; 87.7%) used a mask providing > 90% ultraviolet light protection.

There was no association between the investigated variables (eye affected, breed, sex, age, number of affected sites, histologic diagnosis, margins, sutures placed, sedation versus anesthesia, post‐operative topical chemotherapy, post‐operative UV light protection) and exam reported complications or individual exam reported complications (epiphora, conjunctival adhesion, fat prolapse, enophthalmos). Due to the low number of observations for the individual exam reported complications and suture placement, statistical analysis could not be performed.

**FIGURE 4:**
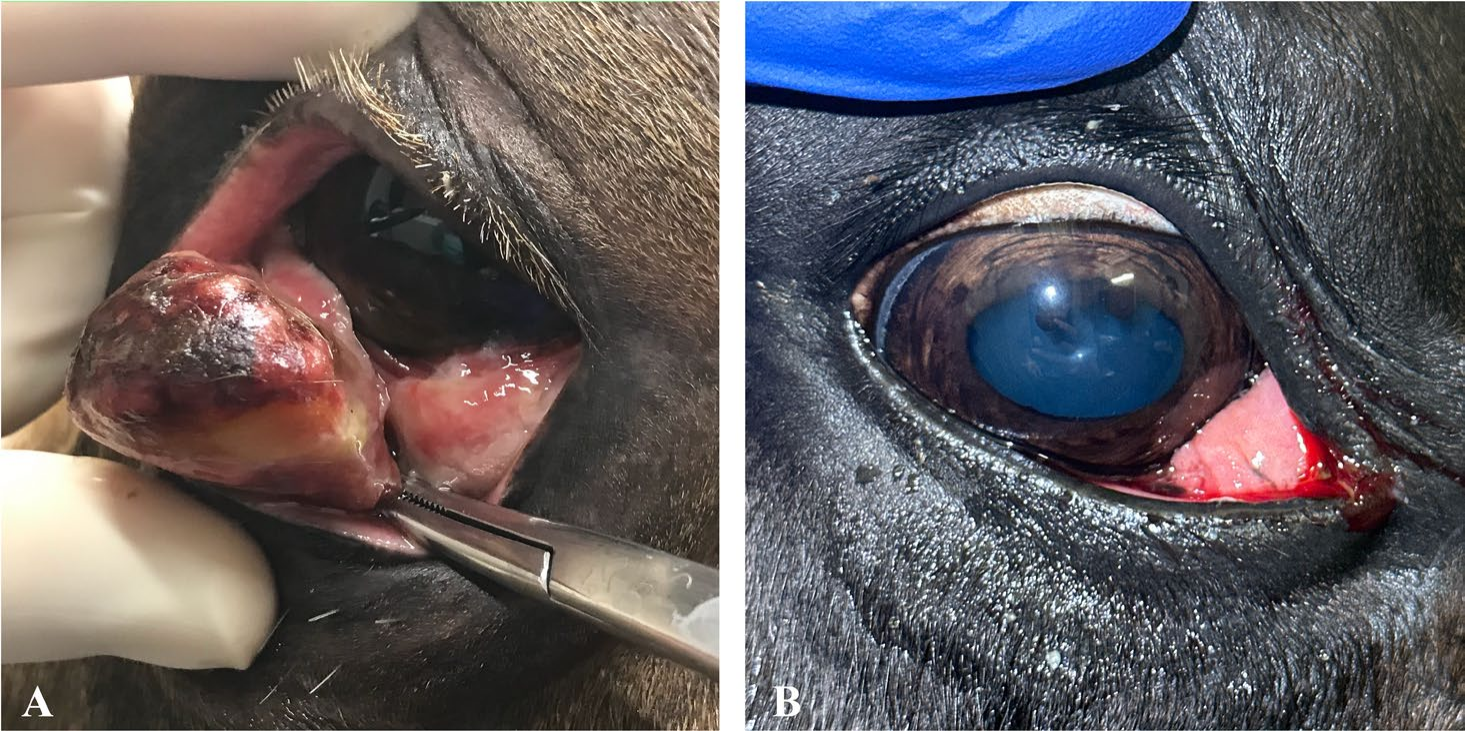
(A) Large fat prolapse and subsequent necrosis at the time of removal, which was 1 week postoperative nictitating membrane resection. (B) Fat prolapse without necrosis 24 h after nictitating membrane resection.

Complete removal of the nictitating membrane was performed in all cases. Standing sedation was used in 77 horses (92.7%), while general anesthesia was used in 6 horses (7.2%). Sutures were placed postoperatively in 12 eyes (13%) and not placed in 80 eyes (87%). The incidence of fat prolapse was low (Figure 4), occurring in 4.3% of cases. There were no reported cases of fat prolapse in horses with sutures placed, though this difference was not statistically significant (owner reported complications *p* = 0.80, exam reported complications *p* = 0.233).

**FIGURE 5:**
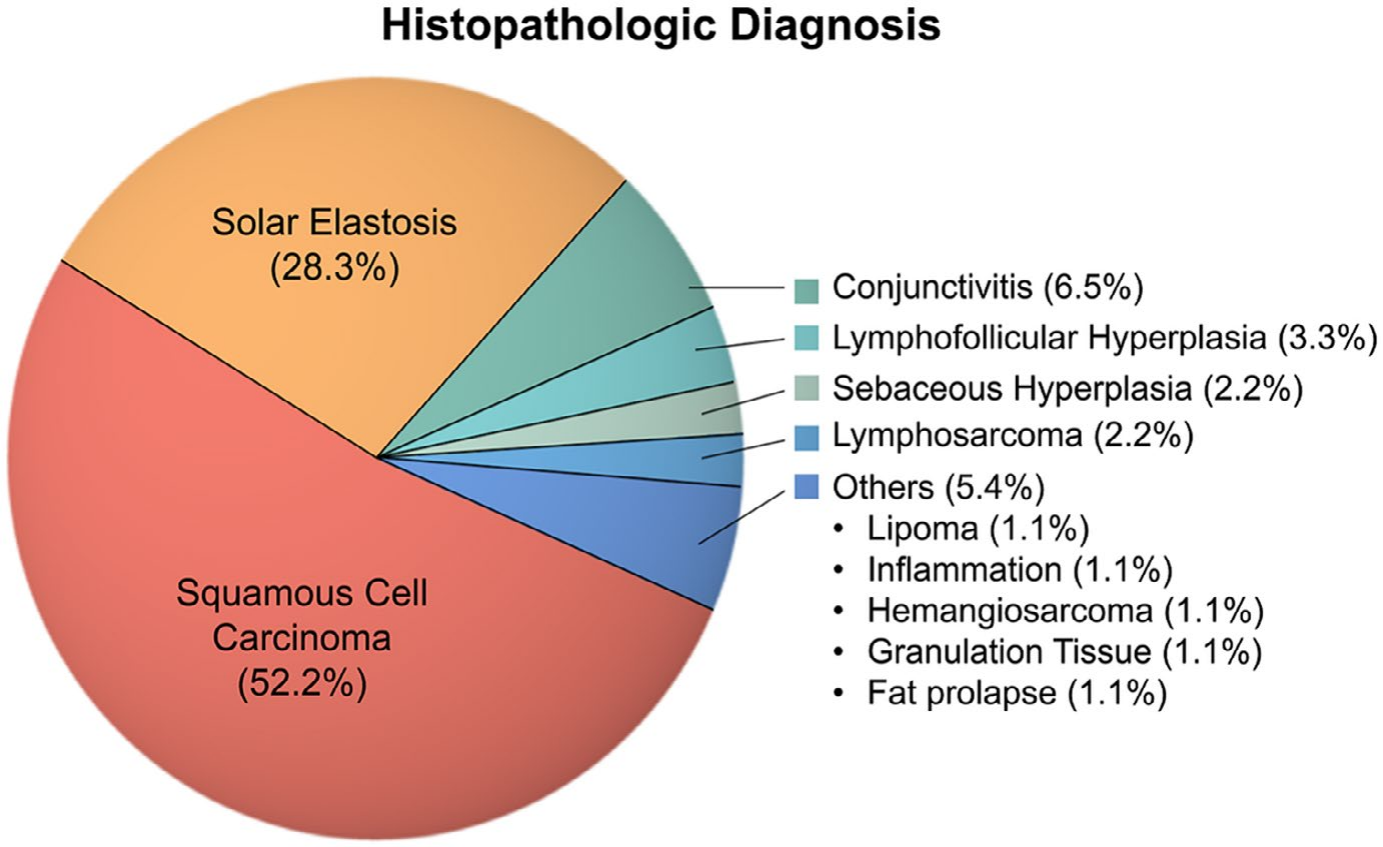
Distribution of histopathologic diagnoses from nictitating membrane resection samples.

Histopathologic diagnosis was performed in all cases (Figure 5). Neoplasia was confirmed in 51 of 92 (55%) eyes; of those cases SCC was diagnosed in 94% (*n* = 48), lymphosarcoma in 4% (*n* = 2), and hemangiosarcoma in 2% (*n* = 1). Non‐neoplastic lesions were diagnosed in 41 nictitating membranes and included solar elastosis (*n* = 26), inflammatory lesions (lymphoplasmacytic and pyogranulomatous conjunctivitis; *n* = 7), hyperplasia (lymphoid follicular and sebaceous hyperplasia; *n* = 5), adipose tissue (*n* = 2), and granulation tissue (*n* = 1). Histopathology was not associated with any complication.

## 4 Discussion

**FIGURE 6:**
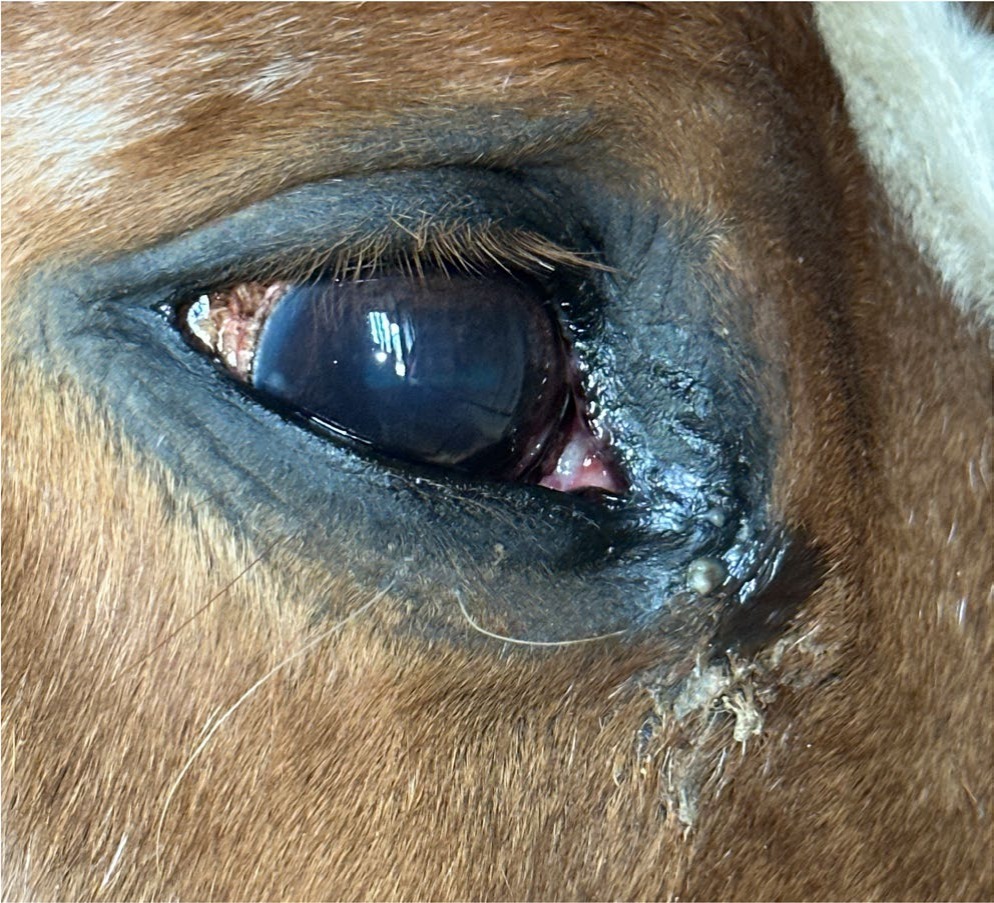
An example of epiphora as a long‐term complication following nictitating membrane resection.

Overall, owner‐reported complications following nictitating membrane resection occurred in 28.2% of eyes, and complications identified on recheck examination occurred in 23.1% of cases; most complications were mild and easily managed. The most common complication after the nictitating membrane resection in the present study was chronic epiphora (Figure 6), reported by owners in 22.8% (21/92) of eyes.

The cause of chronic mild epiphora following nictitating membrane resection is unknown. Loss of the protective function of the nictitating membrane and subsequent accumulation of dust, debris, and mucus within the ventral conjunctival fornix has been proposed in the literature as a contributing factor to chronic epiphora following nictitating membrane resection [11], which we suspect to be the predominant reason. Chronic epiphora should be mentioned as a possible long‐term complication to owners prior to performing the procedure.

Although resection of the nictitating membrane has been linked to an increased risk of developing keratoconjunctivitis sicca (KCS) and corneal opacity in dogs [17], clinical signs of KCS were not reported in the present study. Owners were specifically questioned regarding signs of decreased tear production, including mucoid discharge, conjunctival hyperemia, changes in corneal clarity, and vision status. However, Schirmer tear testing was not performed in this study, and subclinical changes in tear production cannot be excluded. Future prospective studies should include both preoperative and postoperative Schirmer tear testing to determine whether nictitating membrane resection results in quantitative changes in tear production that may not be clinically apparent. In addition, qualitative tear film assessment is recommended to further evaluate potential alterations in tear film stability and ocular surface health.

**FIGURE 7:**
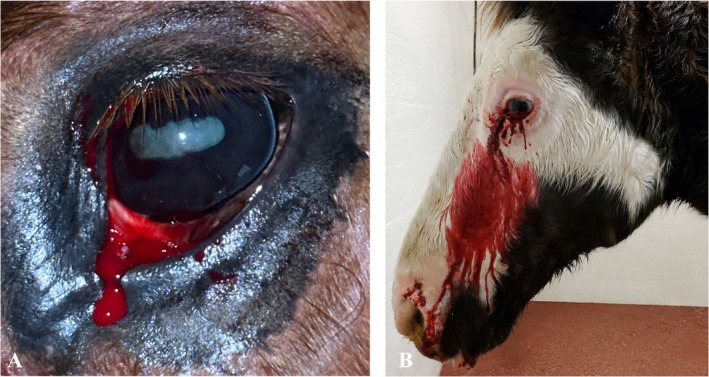
An example of mild hemorrhage (A) and severe hemorrhage (B) immediately following nictitating membrane resection.

The incidence of hemorrhage (Figure 7) in this study was low (1.1%; 1/92); however, after an initial case of postoperative hemorrhage, management of these cases was changed. Standard practice now includes compression over the medial canthus immediately after resection and placing the horse on cross ties with a hay net available in their stall until bleeding completely stops. Elevation of the head following surgery was used to help reduce postoperative bleeding by lowering vascular pressure and improving venous drainage from the surgical field [18]. This appears to help reduce postoperative bleeding and since this management change, horses have had only mild hemorrhage that stops completely within a few hours.

Prolapse of orbital fat has previously been described as a potential complication of nictitating membrane resection in horses [19]. When the nictitating membrane is removed, an opening develops between the conjunctival lining and the orbital fat, allowing fat to protrude into the fornix, where it is susceptible to necrosis [19]. Primary closure of the conjunctival wound has been recommended to minimize the risk of orbital fat prolapse [19]. In the present study, sutures were not placed to close the conjunctival wound in the majority of cases. The incidence of fat prolapse was low (4.3%; 4/92), and there was no statistical difference between eyes with sutures placed and those without sutures. However, it is important to consider that the group that had sutures placed had no cases of fat prolapse (0/12), whereas all cases occurred in the no‐suture group (4/80; 5%).

A notable finding of this study was that neoplasia was histopathologically confirmed in 55% of excised nictitating membranes, despite all cases undergoing surgery for a mass or lesion affecting the nictitating membrane. The presence of non‐neoplastic tissue in many cases highlights the potential value of preoperative diagnostic confirmation. The authors performing nictitating membrane resection did so with the knowledge that some cases would be non‐neoplastic; however, the presence of precancerous changes (28.2%, *n* = 26) in the majority of these cases made resection valuable and hopefully preventative. Given the potential for actinic damage, solar elastosis, and other precancerous lesions to progress or harbor early neoplastic transformation, complete nictitating membrane resection should be considered both diagnostically and therapeutically valuable in clinically suspicious cases. The risk of delayed diagnosis or progression to neoplasia should be weighed against the relatively low and manageable morbidity associated with complete excision. Preoperative biopsy may assist in case selection and surgical planning; however, its use must be considered in the context of practical limitations, including multiple veterinary visits, increased cost, and logistical challenges such as repeated trailer transport. These factors likely contributed to the absence of preoperative biopsies in this cohort. Nonetheless, biopsy may be considered in selected cases with diagnostic uncertainty to aid clinical decision‐making and inform client expectations. It is important to consider that preoperative biopsy has previously been shown to be associated negatively with outcome [12], so in cases of nictitating membrane neoplasia, complete resection as soon as possible is strongly recommended.

Squamous cell carcinoma (SCC) was the most common neoplasm diagnosed in the present study, which is consistent with previous reports [2, 12]. The overrepresentation of Haflingers, Belgians, and other draft‐type breeds in this cohort is consistent with previously reported breed predispositions to ocular SCC [1, 2, 3, 4]. Although referral patterns may have contributed to the observed breed distribution, the findings likely also reflect established genetic and phenotypic risk factors rather than random sampling variation within the hospital population. A missense variant in the damage‐specific DNA binding protein 2 (DDB2) gene has been identified as a significant genetic risk factor for ocular SCC, particularly in Haflinger and Belgian horses [20, 21, 22]. This variant has been shown to account for a large proportion of SCC cases in these breeds [23]. These findings indicate that genetic predisposition, in combination with known risk factors such as lighter or absent periocular pigmentation and ultraviolet light exposure, likely contributes to the increased prevalence of ocular SCC observed in these breeds. Therefore, the breed distribution observed in this study likely reflects underlying genetic and phenotypic predispositions rather than sampling variation.

This study has several limitations including the inherent lack of standardization encountered when performing retrospective research. Although this is the largest group of horses that underwent nictitating membrane resection reported in the literature, the sample size was relatively small which may have limited the ability to detect differences, especially in the categories of complications that were low in number. Another limitation was the reliance on owner feedback for the majority of complication reporting. Prospective studies are warranted to further evaluate these factors. As a goal for future research, complete ophthalmic examinations, including quantitative and qualitative tear testing, should be performed on horses prior to and following nictitating membrane resection to help determine the cause of chronic epiphora.

## 5 Conclusion

Although owner and exam reported complications were seen post‐operatively in one‐quarter of horses that had nictitating membrane resection, complications were mild and easily managed. All owners were satisfied with the outcome of the procedure. Chronic mild epiphora is the most common complication and should be mentioned as a possible long‐term complication to owners.

## Author Contributions

**Nicole M. Scherrer:** conceptualization, writing – original draft, writing – review and editing, supervision, investigation, methodology, validation, resources, visualization. **Tiantian Wu:** writing – original draft, writing – review and editing, data curation, conceptualization, investigation, methodology. **Darko Stefanovski:** formal analysis.

## Disclosure

Artificial Intelligence Statement: The authors have not used AI to generate any part of the manuscript.

## Ethics Statement

This study complies with the Guidelines for Ethical Research in Veterinary Ophthalmology (GERVO) and is exempt from approval by an ethics committee.

## Conflicts of Interest

The authors declare no conflicts of interest.

## Supporting information


**File S1:** Owner follow‐up questionnaire used for horses undergoing nictitating membrane resection.

## Data Availability

The data that support the findings of this study are available from the corresponding author upon reasonable request.

## References

[1] F. C. Blodi and F. K. Ramsey , “Ocular Tumors in Domestic Animals,” American Journal of Ophthalmology 64 (1967): 627–633. 6068464 10.1016/0002-9394(67)90568-5

[2] J. D. Lavach and G. A. Severin , “Neoplasia of the Equine Eye, Adnexa, and Orbit: A Review of 68 Cases,” Journal of the American Veterinary Medical Association 170 (1977): 202–203. 833045

[3] R. E. Junge , J. P. Sundberg , and W. D. Lancaster , “Papillomas and Squamous Cell Carcinomas of Horses,” Journal of the American Veterinary Medical Association 185 (1984): 656–659. 6092315

[4] S. J. Dugan , M. S. Roberts , C. R. Curtis , et al., “Epidemiologic Study of Ocular Adnexal Squamous Cell Carcinoma in Horses,” Journal of the American Veterinary Medical Association 198 (1991): 251–256. 2004985

[5] C. Baril , “Basal Cell Tumour of Third Eyelid in a Horse,” Canadian Veterinary Journal 14 (1973): 66–67. PMC1696058 4691537

[6] H. Gehlen and P. Wohlsein , “Cutaneous Lymphangioma in a Young Standardbred Mare,” Equine Veterinary Journal 32 (2000): 86–88. 10661392 10.2746/042516400777612017

[7] R. L. Mathes , K. P. Carmichael , J. Peroni , K. Paige Carmichael , and P. Anthony Moore , “Primary Lacrimal Gland Adenocarcinoma of the Third Eyelid in a Horse,” Veterinary Ophthalmology 14 (2011): 48–54. 10.1111/j.1463-5224.2010.00840.x 21199279

[8] C. Puff , V. Herder , A. Philipp , and W. Baumgärtner , “Lymphangiosarcoma in the Nictitating Membrane of a Horse,” Journal of Veterinary Diagnostic Investigation 20 (2008): 108–110. 18182523 10.1177/104063870802000124

[9] J. Sansom , D. Donaldson , K. Smith , A. S. Blunden , A. Petite , and M. E. D. Seeliger , “Haemangiosarcoma Involving the Third Eyelid in the Horse: A Case Series,” Equine Veterinary Journal 38 (2006): 277–282. 16706287 10.2746/042516406776866336

[10] A. T. Schnoke , D. E. Brooks , D. A. Wilkie , et al., “Extraocular Lymphoma in the Horse,” Veterinary Ophthalmology 16 (2013): 35–42. 22500697 10.1111/j.1463-5224.2012.01016.x

[11] A. L. Labelle , A. G. Metzler , and D. A. Wilkie , “Nictitating Membrane Resection in the Horse: A Comparison of Long‐Term Outcomes Using Local vs. General Anaesthesia,” Equine Veterinary Journal. Supplement 40 (2011): 42–45, 10.1111/j.2042-3306.2011.00486.x. 22082445

[12] N. M. Scherrer , M. Lassaline‐Utter , and B. C. McKenna , “Characterization and Outcome Following Excision of Masses in the Nictitating Membranes of Horses: 50 Cases (1998–2012),” Journal of the American Veterinary Medical Association 245, no. 7 (2014): 812–815, 10.2460/javma.245.7.812. 25229533

[13] S. J. Dugan , M. S. Roberts , C. R. Curtis , et al., “Prognostic Factors and Survival of Horses With Ocular/Adnexal Squamous Cell Carcinoma: 147 Cases (1978–1988),” Journal of the American Veterinary Medical Association 198 (1991): 298–303. 2004996

[14] S. Kaps , M. Richter , M. Philipp , M. Bart , C. Eule , and B. M. Spiess , “Primary Invasive Ocular Squamous Cell Carcinoma in a Horse,” Veterinary Ophthalmology 8 (2005): 193–197. 15910373 10.1111/j.1463-5224.2005.00358.x

[15] K. N. Gelatt , V. S. Myers, Jr. , V. Perman , and C. Jessen , “Conjunctival Squamous Cell Carcinoma in the Horse,” Journal of the American Veterinary Medical Association 165 (1974): 617–620. 4425154

[16] R. J. Payne , M. S. Lean , and T. R. C. Greet , “Third Eyelid Resection as a Treatment for Suspected Squamous Cell Carcinoma in 24 Horses,” Veterinary Record 165 (2009): 740–743. 20023277

[17] R. V. Morgan , J. M. Duddy , and K. McClurg , “Keratoconjunctivitis Sicca Associated With Third Eyelid Removal in Dogs,” Journal of the American Veterinary Medical Association 202, no. 2 (1993): 261–264.8428832

[18] S. R. Caffey , C. M. Lund , K. D. Farnsworth , B. A. Fransson , and C. A. Ragle , “Effects of Head Position on Internal and External Carotid Pressures in Standing Sedated Horses,” Canadian Journal of Veterinary Research 85, no. 2 (2021): 127–130. 33883820 PMC7995541

[19] E. A. Giuliano , “Diseases of the Equine Adnexa,” in Equine Ophthalmology, 2nd ed., ed. B. C. Gilger (Elsevier/Saunders, 2010), 133–180.

[20] R. R. Bellone , J. Liu , J. L. Petersen , et al., “A Missense Mutation in Damage‐Specific DNA Binding Protein 2 Is a Genetic Risk Factor for Limbal Squamous Cell Carcinoma in Horses,” International Journal of Cancer 141 (2017): 342–353, 10.1002/ijc.30744. 28425625

[21] M. Singer‐Berk , K. E. Knickelbein , S. Vig , et al., “Genetic Risk for Squamous Cell Carcinoma of the Nictitating Membrane Parallels That of the Limbus in Haflinger Horses,” Animal Genetics 49 (2018): 457–460, 10.1111/age.12695. 29999543

[22] K. E. Knickelbein , M. E. Lassaline , M. Singer‐Berk , et al., “A Missense Mutation in Damage‐Specific DNA Binding Protein 2 Is a Genetic Risk Factor for Ocular Squamous Cell Carcinoma in Belgian Horses,” Equine Veterinary Journal 52 (2020): 34–40, 10.1111/evj.13116. 30903710

[23] K. E. Knickelbein , M. E. Lassaline , and R. R. Bellone , “Limbal Squamous Cell Carcinoma in a Rocky Mountain Horse: Case Report and Investigation of Genetic Contribution,” Veterinary Ophthalmology 22 (2019): 201–205, 10.1111/vop.12612. 30238589

