# Supplementary material for: Complications Following Nictitating Membrane Resection in Horses: 92 Eyes (2015–2024)
Source: Vet Ophthalmol. 2026 Aug 6;29(5):e70243. doi: 10.1111/vop.70243 (PMC13445637; doi:10.1111/vop.70243)
Supplement: Supplementary file 1 [file VOP-29-0-s001.docx]

**Supplementary File 1. Owner Follow-up Questionnaire Used for Horses Undergoing Nictitating Membrane Resection**

The following questionnaire was used during telephone or email follow-up interviews with owners of horses that underwent nictitating membrane resection.

**Owner Follow-up Questionnaire**

1. Following surgery, did your horse develop any systemic health problems or long-term medical issues?
   a. Yes
   b. No
   c. If yes, please describe: _______________________________
2. Following nictitating membrane resection, did you observe any ocular abnormalities associated with the surgical eye? Examples may include:
   - Increased tearing (epiphora)
   - Squinting or blepharospasm
   - Conjunctival redness (conjunctival hyperemia)
   - Mucoid ocular discharge
   - Swelling or inflammation
   - Other abnormalities
   a. Yes
   b. No
   c. If yes, please describe: _______________________________
3. If only one nictitating membrane was removed, did you notice any differences between the surgical eye and the contralateral eye following surgery?
   a. Yes
   b. No
   c. If yes, please describe: _______________________________
4. Following surgery, did your horse require an unexpected veterinary examination or treatment related to the surgical eye?
   a. Yes
   b. No
   c. If yes, please describe: _______________________________
5. Did your horse receive postoperative topical or systemic chemotherapy following surgery?
   a. Yes
   b. No
   c. If yes, please specify treatment: _______________________________
6. Did your horse wear a fly mask or ultraviolet (UV)-protective mask following surgery?
   a. Yes
   b. No
   c. If yes, please answer the following questions:
      i. What type of mask was used?
         1. Standard fly mask without UV protection
         2. UV-protective fly mask (<90% UV protection)
         3. UV-protective fly mask (≥90% UV protection)
         4. Custom or specialty ocular protection mask
         5. Unknown
         6. Other: ____________________________________
      ii. How frequently was the mask worn?
          1. Continuously (day and night)
          2. During daytime only
          3. Outdoors only
          4. During summer only
          5. Unknown
      iii. Approximately how long was the mask used following surgery?
           1. Short-term: ________________
           2. Continuously long-term
7. Overall, were you satisfied with the outcome of the procedure?
   a. Satisfied
   b. Neutral
   c. Dissatisfied
   d. Very dissatisfied
8. In your opinion, did the surgery improve your horse’s comfort or quality of life?
   a. Yes
   b. No
   c. Uncertain
   d. Additional comments: _______________________________
9. If the horse was euthanized following surgery:
   a. Was euthanasia considered related to the ocular condition or surgery?
      i. Yes
      ii. No
      iii. Uncertain
   b. Please describe the reason for euthanasia, if known: _______________________________
   c. Approximate date or time interval following surgery: _______________________________
